# Analysis of and function predictions for previously conserved hypothetical or putative proteins in *Blochmannia floridanus*

**DOI:** 10.1186/1471-2180-6-1

**Published:** 2006-01-09

**Authors:** Peter Gaudermann, Ina Vogl, Evelyn Zientz, Francisco J Silva, Andres Moya, Roy Gross, Thomas Dandekar

**Affiliations:** 1 dept of bioinformatics, biocenter university of Würzburg, 97074 Würzburg, Germany; 2 dept of microbiology, biocenter university of Würzburg, 97074 Würzburg, Germany; 3 Departament de Genètica, Institut Cavanilles de Biodiversitat i Biologia Evolutiva de Universitat de València, 46071 Valencia, Spain

## Abstract

**Background:**

There is an increasing interest to better understand endosymbiont capabilities in insects both from an ecological point of view and for pest control. *Blochmannia floridanus *provides important nutrients for its host, the ant *Camponotus*, while the bacterium in return is provided with a niche to proliferate. *Blochmannia floridanus *proteins and metabolites are difficult to study due to its endosymbiontic life style; however, its complete genome sequence became recently available.

**Results:**

Improved sequence analysis algorithms, databanks and gene and pathway context methods allowed us to reveal new information on various enzyme and pathways from the *Blochmannia floridanus *genome sequence [EMBL-ID BX248583]. Furthermore, these predictions are supported and linked to experimental data for instance from structural genomics projects (e.g. Bfl341, Bfl 499) or available biochemical data on proteins from other species which we show here to be related. We were able to assign a confirmed or at least a putative molecular function for 21 from 27 previously conserved hypothetical proteins. For 48 proteins of 66 with a previous putative assignment the function was further clarified. Several of these proteins occur in many proteobacteria and are found to be conserved even in the compact genome of this endosymbiont. To extend and re-test predictions and links to experimentally verified protein functions, functional clusters and interactions were assembled. These included septum initiation and cell division (Bfl165, Bfl303, Bfl248 et al.); translation; transport; the ubiquinone (Bfl547 et al.), the inositol and nitrogen pathways.

**Conclusion:**

Taken together, our data allow a better and more complete description of the pathway capabilities and life style of this typical endosymbiont.

## Background

Genome analysis is improved and becomes more meaningful when one considers genome context, pathway context and metabolic reconstruction. A focus on the prediction of encoded proteins and establishing links to experimentally characterized protein sequences from other organisms is particularly important in organisms where there has been characterized mainly the genome sequence. Examples are obligate intracellular endosymbionts where direct experimental identification of their proteins is very challenging. As a case in point we analyze the genome sequence from the endosymbiont *Blochmannia floridanus*, an obligate intracellular endosymbiont of ants of the genus *Camponotus*. Such symbioses between insects and bacteria are widespread in nature and the endosymbionts are of high importance for their hosts with implications for pest control. *Blochmannia floridanus, Buchnera *and *Wigglesworthia *bacteria provide important nutrients to their insect host [1] and live in intimate metabolic contact [2-6] with the host as intracellular organisms in specialised cells, bacteriocytes [7-9]. The diet of the ant is not as specialized as those of aphids and tsetse flies, the hosts of *Buchnera *and *Wigglesworthia*, respectively, implicating specific metabolic capabilities in *Blochmannia *[10]. However, endosymbiotic genomes are experiencing a steady reduction as the intracellular symbionts live in a constant intracellular environment. The recent genome sequence of *Blochmannia floridanus *[11] allowed first insights in its biochemistry and life style. However, important functions of the proteins encoded in the genome remained uncertain ("putative") or could not yet be recognized ("hypothetical proteins") and are the focus of this study.

**Figure 1:**
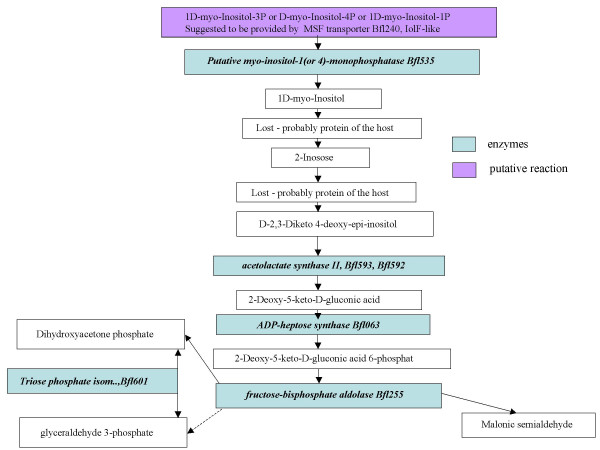
**Inositol Pathway**. The identified predicted enzyme activities of *Blochmannia *are mapped on the pathway of inositol synthesis. Theyinclude: a MFS family transporter (Bfl240, IolF-like, top right; exact substrate specificity not known), ADP-heptose synthase (Bfl063, IolC-like), an acetolactate synthase II, large subunit (Bfl593; IolD-like) and its small subunit, neighbouring protein Bfl 592 (both proteins should physically interact), fructose 1,6-bisphosphate aldolase (Bfl255, IolJ-like; this enzyme is also involved in glycolysis) and predicted myo-inositol-1(or 4)-monophosphatase (Bfl535; related to the Archaeal fructose-1,6-bisphosphatase and related enzymes of inositol monophosphatase family). Bfl601 (triosephosphate isomerase) finishes the pathway converting created dihydroxyacetone phosphate into glyceraldehyde 3-phosphate.

**Figure 2:**
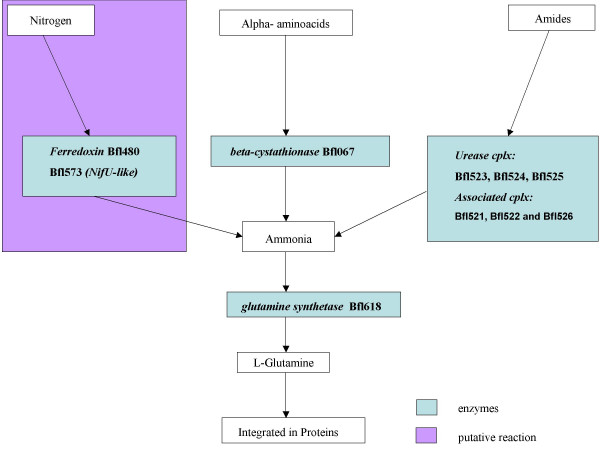
**Nitrogen metabolism**. The identified previously known and newly predicted enzyme activities of *Blochmannia *are compiled for the pathway of nitrogen metabolism. They involve the urease complex (Bfl523, Bfl524 and Bfl525; catalyzing the following reaction: Urea + H_2_O = CO_2 _+ 2 NH_3_) and urease activating complex (Bfl521, Bfl522 and Bfl526) and glutamine synthetase (Bfl618). Further proteins adding to the pathway are the now confirmed ferredoxin (Bfl 480), Bfl 573 (NifU-like protein; previously conserved hypothetical) as well as beta-cystathionase (Bfl067).

The continuous growth of sequence data together with modern sequence alignment algorithms allow to detect homologies to well characterized proteins from other species which have been overlooked by previous efforts. Any pathway in *Blochmannia floridanus *has to be sufficiently complete to fulfil its biological function (in particular to produce a required end product). Furthermore, the full genome sequence of *Blochmannia floridanus *is known. All these factors help in genome-wide sequence comparisons to identify a missing enzyme activity for a *Blochmannia floridanus *pathway. Comparisons of the pathway completeness with other species and biochemical data further refine such a "pathway alignment" [12]. Two more complete pathways obtained here for *Blochmannia floridanus *are shown in the figures (Fig. 1, Fig. 2; colour versions see Additional files 3 and 4).

Genome context methods are a further independent method to deduce sequence function [13]. Clearly, genes which are together in an operon are linked by the common function of the operon. This helps to assign function to sequences in such operons. Comparing co-occurrence or common absence of gene sets in different genomes further enhances this: If an unknown gene is always present with a set of known genes its function is associated with this set of known genes. Furthermore, we could show that proteins found in several prokaryotic genomes to be encoded by neighbouring genes tend to interact with each other [14]. If in one genome such neighbouring reading frames are found to be fused, this is an even stronger indication that the encoded proteins interact (as these open reading frames are even translated into a single protein in this genome). When many genomes are compared in this way for the conservation and variation of genes, genome order and fusion, the method becomes quite powerful [15]. Thus we used for this type of predictions a recent version of the database STRING which compares more than 100 genomes for these various criteria and includes in addition experimental and text mining data on protein-protein interactions.

Combining these different methods allowed a number of new sequence assignments. Error thresholds and combination criteria are indicated in the Materials and Methods section.

## Results

A total of 631 genes and of these 583protein-coding genes are present in the *Blochmannia *genome. According to the recently published complete genome sequence [11], 26 are conserved hypothetical proteins and in this way their assignment points to functions occurring in other species, either other endosymbionts or more general ones. Another protein encoded in the genome was considered hypothetical, presumably species specific and of unknown function but is now found to be present also in other genomes and to be involved in transcription (Bfl390, *yqeI*). Furthermore 66 proteins had only a putative function assignment. These different proteins and their encoding gene sequences were analyzed using various bioinformatical tools (see Materials and Methods).

### Proteins with previous putative function assignments

For the proteins encoded in the complete genome which were termed „putative", we first checked whether we could either further confirm or more solidly describe their function by the additional experimental and bioinformatical data gathered in databases and genomes since the first annotation. Furthermore we investigated with detailed sequence analysis these putative proteins whether we could find more information about their molecular function either by specific motif searches (profiles and motifs known to be critical for specific protein functions from experimentally characterized proteins) or by establishing links (in particular by sequence similarity, domain conservation, clusters of orthologous genes as well as genome context methods) to experimentally well characterized protein sequences in other genomes.

Additional file 1 shows that using sequence to sequence comparisons, an informative assignment with high confidence (e-value below 10^-6^) on the molecular function (categorized as "good"; right column in Additional file 1) of 39 proteins could be made and for 9 proteins there remained only minor uncertainties on the molecular function despite the low e-value of their sequence assignment (categorized as "fair"). For 15 proteins there is still only a putative function assignment possible and for one protein no ("unknown") prediction is possible. Furthermore, in all the cases categorized as "good" and most of those categorized as "fair" the function could also be supported by several methods and databases we used (see Materials and Methods). These allowed to clarify the function of the *Blochmannia floridanus *sequence and established strong links to experimental well characterized protein sequences.

The revised cellular and molecular functions of 64 putative proteins are summarized in Additional file 1. Two pseudo-genes were not included. As an example, Bfl623 protein can now be assigned by sequence similarity to the rmuC family. Experimentally well characterized proteins encoded by this family are involved in limiting inversions (in genetic rearrangements). Furthermore, the sequences of these proteins show limited homology to myosins and to some of the SMC (structural maintenance of chromosomes) proteins [16].

### Pathway additions from the previous putative proteins

Nothing in a genome makes sense if not considered and validated by its interrelations and context [17]. To validate our predictions further and to confirm them in the light of previously recognized functions of encoded proteins, we considered the genome and metabolic context (see Materials and Methods) of the re-analyzed previously putative proteins. The following pathways thus emerge for *Blochmannia*:

Bfl240 (putative MFS family transporter, IolF-like), Bfl063 (ADP-heptose synthase; IolC-like), Bfl593 (acetolactate synthase II, large subunit; IolD-like; small subunit is neighbouring Bfl 592, high certainty that both proteins interact) and Bfl255 (IolJ-like, fructose 1,6-bisphosphate aldolase, this enzyme is also involved in glycolysis) are all predicted to be involved in the **inositol pathway **(Figure 1; colour version in Additional file 3). A further component of this pathway and confirmed in this assignment by the KEGG database is Bfl535 (E.C. number 3.1.1.25) which would be a putative myo-inositol-1(or 4)-monophosphatase related to the Archaeal fructose-1,6-bisphosphatase and related enzymes of inositol monophosphatase family.

Furthermore, we could assemble the following enzymes for **Vitamin B synthesis: **Bfl539 (PdxJ) is involved with erythronate-4-phosphate dehydrogenase (Bfl497), phosphoserine aminotransferase (Bfl383), threonine synthase (Bfl113), pyridoxal phosphate biosynthetic protein PdxA (Bfl127), pyridoxamine 5'-phosphate oxidase (Bfl370). This addition to the original annotation has also been confirmed by the KEGG database.

Regarding **nitrogen metabolism**, we were able to confirm that the encoded proteins Bfl523, Bfl524 and Bfl525 annotated previously as putative [11] build an urease complex (catalyzing the following reaction: Urea + H_2_O = CO_2 _+ 2 NH_3_). Furthermore, there are Bfl521, Bfl522 and Bfl526 to build an accessory complex (Figure 2; colour version in Additional file 4), which activates the urease [11]. Moreover, to utilize the produced ammonia for L-glutamine production, glutamine synthetase is present in *Blochmannia *(Bfl618). Besides protein synthesis, the amino-group of glutamine can be used for synthesis of other amino acids in host and symbiont as well as for aminosugars and nucleotides. However, there is no functional glutaminase known in the *Blochmannia *genome. We only found the conserved region of glutaminases to have low similarity to Bfl528. Furthermore, there is ferredoxin (Bfl480). A conserved hypothetical protein (Bfl573) with a NifU domain is probably involved in nitrogen utilization in nitrogen fixing bacteria and plants. However, we suggest that in *Blochmannia *it supplies a redox-activity (iron-sulfur cluster enzyme, confirmed by several databanks and sequence analysis) involved in nitrogen utilization.

Bfl620 (putative oxidoreductase) is predicted by us to be involved in the **ubiquinone pathway**. Potential interaction partners from the genome could be a series of proteins: Bfl628 (a putative methyltransferase), Bfl481 (NADH dehydrogenase I chain N), 4-hydroxybenzoate octaprenyl transferase (Bfl025), 3-octaprenyl-4-hydroxybenzoate carboxy-lyase (Bfl375), ubiquinone biosynthesis protein (Bfl621), 2-octaprenyl-6-methoxyphenol hydroxylase (Bfl259), ubiquinone/menaquinone biosynthesis methyltransferase UbiE (Bfl622), putative monooxygenase (Bfl318), 3-demethylubiquinone-9 3-methyltransferase (Bfl477); all these functional associations are not only suggested by interaction analysis applying the STRING software, but are also supported by the KEGG database comparing different proteobacteria.

The putative methyltransferase Bfl628 is furthermore predicted to be involved in amino acid metabolism (histidine / tyrosine / tryptophan metabolism, aminophosphonate / selenoamino acid metabolism).

Several of the putative proteins we identified to be **transporters. **These could be confirmed in their main function (transport), using HMMER searches from the respective transporter families on the whole proteom (as predicted from the genome sequence). Nevertheless the suggested substrate specificity still needs further experimental confirmation: Bfl012 (preprotein translocasepreprotein translocase subunit YidC), Bfl024 (inorganic phosphate transporter), Bfl029 (sodium/hydrogen exchanger protein; nearly identical to *E.coli *YjcE and conserved in *Buchnera*), Bfl040 (manganese transport system ATP-binding protein), Bfl041 (manganese transport system permease protein), Bfl240 (MFS family transporter), Bfl444 (Membrane protein TerC), Bfl455 (membrane transporter), Bfl575 (inner membrane protein).

Though there are several transporters, the HMMER searches confirmed that there is only one channel in the *Blochmannia *genome: Bfl073, a membrane protein.

### Previously conserved hypothetical proteins

We used sequence, domain and gene context analysis (see methods) to further elucidate the function of the genome encoded 26 conserved hypothetical and the one previous hypothetical protein. Their conservation in several genomes further supports the functional importance of these reading frames. Furthermore, we tried to establish links (in particular by sequence similarity, domain conservation, clusters of orthologous genes as well as genome context methods) to experimentally better characterized protein sequences from other genomes. Moreover, for some predicted proteins, there are links to experimental data available using homologous structures from structural genomics projects, e.g. for Bfl341 and Bfl499.

The following predicted proteins remained nevertheless unclear: Bfl064, Bfl258, Bfl310, Bfl377, Bfl419, Bfl460. However, recent data by Doerks et al. [18] suggest functions for the previously uncharacterized proteins encoded by the clusters of orthologous groups of proteins COG2960 and COG2835. Bfl064 protein is similar to the sequences encoded in the cluster COG2960 which suggests for Bfl064 having a functional participation in protein biosynthesis. Bfl377 is similar to the sequences encoded in COG2835, and for the proteins encoded by COG2835 a functional participation in lipopolysaccharide biosynthesis is predicted.

For the remaining proteins, sequence comparisons allowed to assign them to specific protein families. We relied only on well described protein families described in high quality protein databases such as the databases COG [36], Pfam [38] and GO [43]. For some proteins, direct predictions from sequence analysis were possible such as prediction of transmembrane helices and specific residues or protein motifs. Additional file 2 summarizes these different predictions. Proteins encoded by the *Blochmannia *genes *bfl165, bfl316, bfl341, bfl390, bfl499 *and *bfl547 *were assigned to specific protein families. These functions, predicted by screening for most similar protein sequences using the sequence alignment algorithm BLAST [19] and the non redundant database at the NCBI, could further be confirmed by predicted functions according to the databases COG and PFAM. Those encoded from the two genes *bfl048 *and *bfl248 *could initially only be assigned to their functional categories combining these different resources.

Further we have been able to assign putative new functions by additional sequence and domain analysis for the following eleven previously conserved hypothetical proteins: Bfl043 is probably an organic solvent tolerance protein. Its sequence is similar to the entry COG1934 of the COG database where no function is assigned. Further sequence comparisons indicated then that the region 34–162 is significantly similar (e-value lower then 10^-6^) to the OstA protein from *E.coli*. In *E.coli *recent experimental data show [20] that the OstA protein is known as an organic solvent tolerance protein and so this prediction for Blochmannia was derived. Similarly, Bfl045 should have transcription regulator activity (COG5007, sequence similarity link to experimentally confirmed BolA-like proteins) and there should be methyltransferase activity for Bfl052 protein (COG 0313, similarity of Bfl052 to the putative tetrapyrrole methylase family protein in *Y.pestis*, there are experimentally confirmed tetrapyrrole methylase proteins in this protein family). Sequence analysis finds a Hes-B like domain in Bfl155 (COG0316, YadR). This may indicate a potential function in nitrogen metabolism. Bfl220 may be a dsRNA binding protein (YrdC domain found, there is similarity to a putative dsRNA-binding protein in *Salmonella typhimurium *LT2); a nudix domain points to a hydrolase activity for Bfl264; the protein Bfl363 is probably involved in Fe-S metabolism and export (contains a Fe-S metabolism associated domain), Bfl367 has electron transporter activity (note a glutaredoxin domain region 18 to 99); Bfl423 is predicted to contain a Zn-dependent hydrolase (including possibility to be a glyoxylase); for Bfl442 one can predict an O-sialoglycoprotein endopeptidase activity (a similarity to a glycoprotease family is apparent), and a metalloendopeptidase activity for Bfl451. Interestingly, the last protein contains a transmembrane domain (the region 195 to 449 is similar to outer membrane proteins related to metalloendopeptidases which are implicated in cell envelope biogenesis).

In a third step, the following proteins were examined in more detail to suggest specific molecular or cellular functions:

Bfl048 (gene *yhcB*) of previously unknown function [11] is a member of a cluster of orthologous genes (the COG number 3105). Sequence analysis allows the following predictions on its function: The protein is predicted to contain a domain with a transmembrane region (residues 2 to 24), three low complexity regions (residues 4–22; 23–38 and 90–110) and a coiled-coil region (residues 27–56). More specifically, according to the Pfam database, the protein sequence can be placed into the DUF1043 family. The function assignment of the DUF1043 family is not clear in the Pfam database. However, considering the transmembrane domain prediction and suggested STRING predictions (interactions for Q8ZB59, a *Yersinia *protein, which is homologous to and finds back Bfl048 in blast comparisons) both the *Yersinia *and the *Blochmannia *protein are putative membrane proteins. Predicted associations of the Q8ZB59 *Yersinia *protein further involve a putative exported protein and an adenylate cyclase. This putative function assignment for Bfl048 is further supported by interactions known for the corresponding *E.coli *proteins. Therefore we suggest here that Bfl048 functions as a periplasmic membrane protein.

**Bfl155 **(gene *yadR*) contains a HesB-like domain including the prosite pattern. Its similarity to known structures allows a structure prediction applying Swiss-Model (data not shown).

The exact function of Bfl165 (gene *ygbQ*) was earlier described as unknown [11]. We now suggest that Bfl165 protein functions as a septum formation initiator. Clusters of orthologous genes (COG2919) confirm the septum formation initiator function by looking what is known about the function for orthologous genes of *ybgQ *in other genomes. Thus the homology to the *Yersinia pestis *protein sequence supports the finding that this is with high probability a cell division FtsB protein (DivIC) homolog. Further analysis by sequence comparison finds an amino-terminal coiled-coil domain according to the Pfam database. Moreover, a septum formation initiator domain is predicted for the regions 9 to 99 of the Bfl165 protein by the conserved domain server. We note that in *Blochmannia *several further Fts proteins are predicted to be present ([11] and direct sequence analysis): FtsJ (23S methyltransferase involved in cell division) and Fts A,H,K,L,I,Q,W and Z. The pathway for cell division is only sufficiently complete to allow cell division if also Fts B is present (at least according to genetic data from *E.coli*) and thus this function prediction also closes a gap in an otherwise incomplete pathway in *Blochmannia*.

For the **Bfl220 **protein (gene *yrdC*) the functional assignment is a putative translation factor. The gene encoding the yeast homolog *sua5 *was identified as a suppressor of a translation initiation defect in cytochrome c and is required for normal growth in yeast. The sequence homology of Bfl220 could be confirmed by sequence similarity using different algorithms and databanks as well as by the functional associations predicted for other YrdC proteins. Moreover, in our structure prediction for Bfl220 (by homology modelling as there is a known structure template, the same gene product from *E.coli*, YrdC, brookhaven code 1HRU) the protein features a large concave surface on one side that exhibits a positive electrostatic potential. The authors of the *E.coli *structure suggested that the fold plays a role in double-stranded RNA binding [21].

**Bfl248 **protein (gene *yggX*) was also assigned with an "unknown" function [11]. It was described as a conserved hypothetical protein and with COG category S (Function unknown) which is in line with the Pfam and Interpro (DUF495 family) function assignment. With the growth of function assignment in the COG database COG2924 yields now the putative function assignment Fe-S cluster protection protein. Further sequence analysis supports this. The sequence (Additional file 1) has homology to the PIRSF029827 family, the Fe(II) trafficking protein YggX. The protein represented by this family, YggX, serves to protect Fe-S clusters from oxidative damage [22]. The effect is two-fold in that the proteins which rely on Fe-S clusters do not become inactivated, and the release of free iron and hydrogen peroxide – a DNA damaging agent – is prevented. These observations are consistent with the hypothesis that YggX chelates free iron, and recent experiments show that YggX can indeed bind Fe(II) *in vitro *and *in vivo *in *Salmonella enterica *[22]. Furthermore, YggX has a positive effect on the action of at least one Fe(II)-responsive protein. The combined actions of YggX is reminiscent of iron trafficking proteins and YggX is therefore proposed to play a role in Fe(II) trafficking. In *Escherichia coli*, YggX was shown to be under the transcriptional control of the redox-sensing SoxRS system [23], which is absent in *Blochmannia*.

**Bfl264 **(gene *ygdP*) is a NTP pyrophosphohydrolase confirmed also by data from PFAM and Prosite. Such activities are found in oxidative damage repair enzymes. No potential interactions could be predicted for the protein. Structural homology allows to predict its structure using the solution structure of the nudix enzyme diadenosine 5',5"'-P1, P4-tetraphosphate hydrolase from *Lupinus angustifolius *as the modelling template (Protein databank coordinate file 1F3Y).

**Figure 3:**
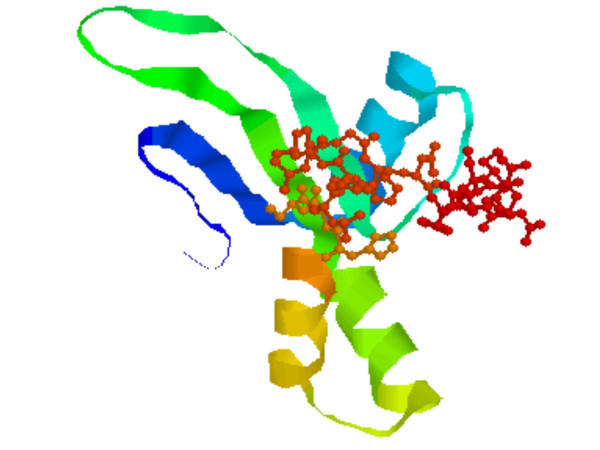
**Homology model of the predicted hydrolase Bfl316. **(template:1OZ9.pdb; hypothetical protein Aq_1354 of *Aquifex aeolicus*). The model starts at residue 33, a phenylalanine. The family signature (UPF, residues from 113 to 130 in Bfl316) is indicated in ball-and-stick. Note that in the template 1OZ9 from pdb databank the structure coordinates are exactly known, however the protein function explained in results was not previously apparent.

**Bfl316 **(gene *ybeY*) was previously classified as functionally unknown (COG0319 and COG Category S; [11]). Function prediction of a COG with previously unknown function profits from any experimental evidence for the function found in orthologous genes in other genomes. In the time after the original publication [11] of the *Blochmannia floridanus *genome sequence, it was revealed for the COG0319 that such genes encode probably a metal-dependent hydrolase (COG0319 and hence the COG is now listed in category R). Independently, from the database ProDom the sequence is predicted to be a metalloenzyme. According to the database Pfam it has the UPF0054 signature with three conserved histidines at the C terminus. In the latter database, all UPF protein family members have unclear function. However, the signature shows which residues are important in this family, in this case the three histidines at the C-terminus. Taking the information from COG database, Prodom and Pfam together predicts for Bfl316 already that it is a metalloenzyme, a hydrolase requiring the metal for catalysis and having three functional important and conserved histidines at the C-terminus. In the course of a structural genomics project on *Aquifex aeolicus *the *A. aeolicus *protein orthologous to Bfl316 was crystallized. Its structure was solved, though its function was not yet known. This allowed us to obtain (see Materials and Methods) a homology model for Bfl316 using this *A.aeolicus *structure as a template. Its predicted structure is shown in Figure 3 (colour version in Additional file 5). The RMSD to the template structure 1OZ9 is 2.49 Å. As the analysis above now gives an indication for its putative function, we offer now both structure information and the putative function for this protein. Furthermore, there is even a prediction possible for a potential interaction partner of this *Blochmannia *protein. Gene context analysis using the STRING database (see Materials & Methods) suggests the functional association of Bfl316 with the PhoH-like protein (**Bfl317**). We have to stress that each of these prediction types relies on independent methods and several databases were independently used. Furthermore, the STRING prediction does not only look at the operon context of *Blochmannia *but makes an estimate and prediction for functional association and interactions comparing well over 100 genomes, most of them prokaryotic.

**Figure 4:**
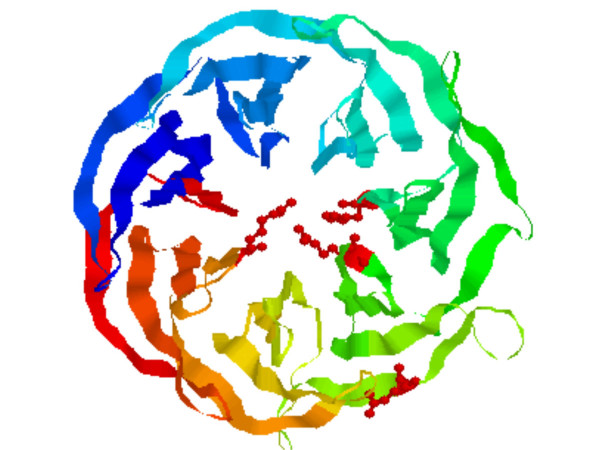
**Homology model of Bfl341 protein**. (template:1RI6; putative isomerase YbhE of *E. coli*). The β-propeller found in 1RI6 [24] is also evident in the homology model (starting from residue 1, a Met in Bfl341). Important predicted catalytic residues outline the substrate cleft. These are His 130, Arg 180, Glu 217 and Arg 281 of Bfl341 corresponding to His 148, Arg 196, Glu 212, and Arg 274 in the template structure (four from five conserved residues known for the family and those checked from *N.crassa *by mutagenesis to be important for catalysis in vitro [24]). Kajander et al. [24] did not know yet the accurate molecular function of the *E.coli *structure they solved. However, regarding the high sequence similarity of Bfl341 (38% identical, 57% similar residues) to recently characterized *E.coli *Pgl protein [25], the actual activity of Bfl341 we now predict to be a 6-phosphogluconolactonase.

**Bfl341 **(gene *ybhE*) was also annotated with unknown function [11]. It was originally described as a conserved hypothetical protein with COG2706 and COG Category R (General function prediction only). With the growth of the COG database one can now infer that the protein clusters into the 3-carboxymuconate cyclases and has the new Category is G (Carbohydrate transport and metabolism). Such an activity would indicate a 3-Carboxy-cis,cis-muconate lactonizing enzyme (EC 5.5.1.5). Muconate lactonizing enzymes (MLEs) convert cis,cis-muconates to muconolactones in microbes as part of the beta-ketoadipate pathway; some also dehalogenate muconate derivatives of xenobiotic haloaromatics. There are three different MLE classes unrelated by evolution, this one belongs to the bacterial CMLE with sequence similarity to class II fumarases and structurally similar to the G protein beta subunit. This allows homology modelling (see Materials and Methods) of Bfl341. The predicted structure for the cycloisomerase is a beta-propeller ([24], Figure 4; colour version in Additional file 6). The RMSD of the Bfl341 homology model to its template structure 1RI6 is 0.25 Å. However, as the sequence similar YbhE protein from *E.coli *has just been confirmed by biochemical experiments to be phosphogluconolactonase (gene *plg*) in the pentose phosphate cycle [25], we can conclude with more confidence that this should be the same activity in *Blochmannia*.

**Bfl390 **(gene *yqeI*) was unknown at the time of the genome annotation. It was declared as a hypothetical protein with COG3710 and COG Category S (Function unknown). Now we find that it contains a DNA-binding winged-HTH domain (CadC, it is assigned COG3710 and COG Category K (Transcription). This could be confirmed as the protein contains a Trans_Reg_C domain (amino acids 27–104, according to Pfam, Interpro; this part of the structure is known in homologues in full atomic detail: 1GXP, 1GXQ, 1ODD are different protein data bank coordinate sets for this). C-terminally (amino acids 149–171) a transmembrane helix is present. Taken together these results suggest that this protein may switch on and off transcription in response to a further signal, which may be a membrane signal detected by the transmembrane domain. There is no receiver domain detectable nor are any two component systems detected as individual components in the *Blochmannia *genome using HMMER search with SMART families on the complete genome.

**Figure 5:**
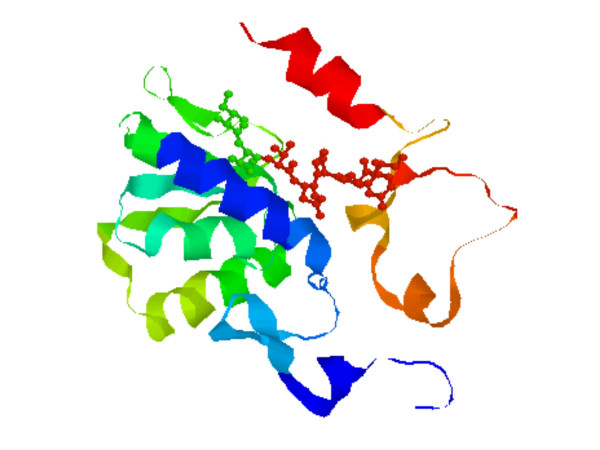
**Homology model of Bfl499 protein**. (template:1NV8; HemK Protein from *Metanococcus jannaschii*). Sensitive sequence alignment algorithms predict this protein to be a N-6 adenine specific DNA methylase or general S-adenoslmethonine-dependent methyltransferase by similarities to experimentally well characterized proteins such as the close relative *E.coli*. Moreover, for the homologue in *M.jannaschii *the three dimensional structure is known. This allows to establish the homology model shown here. Details of the structure: The N6-methylase prosite motif IItNPPY, residues 121 to 127, is highlighted in ball and stick. The fold consists of 3 layers, a/b/a; forming a mixed beta-sheet of 7 strands, order 3214576; strand 7 is anti-parallel to the rest. The homology of the Bfl499 protein does not include the additional helical domain present in 1NV8.

Protein **Bfl499 **(gene *yfcB*) was described as a conserved hypothetical protein. Sequence analysis with searches in current non redundant sequence databases show now that it is a predicted rRNA or tRNA methylase. These could be further confirmed by data from the databanks Interpro and TIGR. The N-methyltransferase activity, also apparent by comparative genome analysis could either use DNA or RNA as a substrate, both is possible for this HemK family member (N-6 adenine specific DNA methylase or general S-adenoslmethonine-dependent methyltransferase as in the close relative *E.coli*). To further support this we find that the sequence is an orthologue of the COG2890 group of genes which have HemK family as functional annotation of the encoded protein. There is a HemK family member with experimentally solved protein structure in *Metanococcus jannaschii*. This allowed us to obtain (see Materials and Methods) a homology model for Bfl499. The predicted structure is shown in Figure 5 (colour version in Additional file 7). The RMSD to its template structure 1NV8 is 0.59 Å.

**Bfl547 **protein (gene *yfjG*) was unknown (category S and COG2867 family). Now it can be shown to be an oligoketide cyclase and with this function it may be involved as a lipid transport protein (prediction according to COG). Pfam confirms that it belongs to Pfam family PF03654, the Aromatic-Rich Protein Family. This family may be related to polyketide synthases. The synthesis of polyketides in *Blochmannia *shows a more complex lipid metabolism. In *Blochmannia*, polyketides can furthermore be involved supporting the lipid metabolism in the host, in particular regarding insect hormones.

The **phylogenetic distribution **of the previously conserved hypothetical proteins is shown in Table 1. Interestingly, there are no *Blochmannia *specific proteins among them, even the protein Bfl390 is found in *Buchnera *and *Wigglesworthia*. On the other hand, the conserved hypothetical protein Bfl310 belongs to a wide-spread family found in many non-proteobacteria. This is also true for the following proteins with some functional assignment: Bfl052, Bfl155, Bfl220, Bfl367 and Bfl423.

### Predicted functional associations and interactions for the previously conserved hypothetical proteins

The tool STRING [13,26] allows to predict potential functional associations (including cases with direct physical interaction) for the new annotated proteins by comparison of conserved gene neighbourhood, gene fusion, common absence or presence as well as homology to proteins with experimentally characterized interactions in different bacterial and other genomes. One has to stress that these are only potential associations, which need further experimental confirmation. However, these predictions have been shown to be robust [13,26] for the delineated functional clusters, genes associated by a common physiological function, even if an individual interaction turns out to be wrong in subsequent experiments. Only predicted partners present in the *Blochmannia *genome are considered in the following.

No potential interactions were predicted for the genome encoded proteins Bfl390 and Bfl341.

The periplasmic protein **Bfl048 **is associated and may functionally interact with the putative exported proteins **Bfl090 **and **Bfl279 **(the latter with the Pfam Bac_surface_Arg signature) as supported by predictions applying the STRING tool [13]. These conserved proteins are all involved in membrane formation, and may perhaps in this sense also participate in cell division.

The septum initiation formation FtsB-like protein **Bfl165 **is tentatively functionally associated with the enzyme **Bfl303 **(an UDP-2,3-diacylglucosamine hydrolase with metallophos-like regulation and suggested cellular function in membrane glycosilation) as well as with the N-methyl-transferase activity for DNA and possibly also for RNA substrates of **Bfl499**. Furthermore it is functionally associated with **Bfl248 **protein, for which a function is difficult to assign (protein family is Pfam DUF495 with unknown molecular function) and **Bfl157**, an enolase (EC 4.2.1.11), most probable a 2-phosphoglycerate dehydratase).

Functional associations predicted from other genomes suggest also for the Bfl248 protein a functional involvement in the synthesis of complex sugars and nucleotide synthesis during DNA synthesis and cell wall septum initiation in cell division. The neighbouring gene *bfl249 *encodes a DNA binding purine specific glycosylase using an iron-sulphur binding domain. This points to an endonuclase III-like DNA glycosidase and a cellular function as a DNA repair protein. Further functional associations using the STRING interaction database suggest a function in nucleotide metabolism for Bfl248. There are two cellular functions predicted for Bfl248 protein: **Bfl248 **is predicted to be associated with **Bfl311**, the DNA polymerase III delta subunit, the Bis(5'-nucleosyl)-tetraphosphatase of **Bfl125**, as well as with **Bfl368 **(Ribonuclease T-activity), the membrane-bound ATP synthase F0 sector subunit A **Bfl002**, and the tRNA nucleotidyl-transferase acitivity of **Bfl062**.

The *Bfl316 *gene has been considered essential based on its presence in distant reduced genomes, such as proteobacteria and Mycoplasma [27]. The encoded metal dependent hydrolase Bfl316 is furthermore predicted to be functionally associated with Bfl317, a cytoplasmatic protein and predicted ATPase that is induced by phosphate starvation. Furthermore, as may be expected from the molecular function of Bfl316, it is predicted to be functionally associated with the magnesium and cobalt efflux CorC-like transporters **Bfl315 **and **Bfl444**. Further less strong associations are found for ribosomal proteins L9 **Bfl087**, and L20 (**Bfl354**) and the RNA polymerase sigma 70 domain **Bfl626 **(a heat shock protein, RNA polymerase sigma 32 factor) suggesting a role of the hydrolase in translation.

**Bfl499 **protein is predicted to be associated with putative cytoplasmic protein (hypothetical protein STY3216 with Pfam DUF710) **Bfl258**.

Besides the previously identified polyketide cyclase **Bfl405 **we identify now **Bfl547 **as an oligoketide cyclase. Its trans-acetylation activity is predicted to be associated with several *Blochmannia *genes for the ubiquinone pathway (3-demethylubiquinone-9 3-methyltransferase, Ubi E-like **Bfl622**; 4-hydroxybenzoate octaprenyltransferase, UbiA-like **Bfl025**; the monooxygenases **Bfl318 **and **Bfl259 **(2-octaprenyl-3-methyl-6-methoxy-1,4-benzoquinol hydroxylase) as well as the succinate dehydrogenase cytochrome b-556 subunit **Bfl327**. This may suggest that **Bfl547 **trans-acetylates succinyl-CoA or some of the lipids involved in ubiquinone synthesis. We suggest further that the protein may be involved in the exchange and trans-membrane-transport of metabolites to or from the host.

## Discussion

### Reannotation of prokaryotic genomes

Our study combines a number of bioinformatics tools for function predictions of previously not assigned proteins in an endosymbiont genome where currently direct data on proteins are difficult to obtain. We have to stress that besides sequence analysis with latest sensitive alignment algorithms we combine here the latest versions of several protein family databases, protein motifs, intrinsic features from the amino acid sequence as well as pathway and genome context methods. All these methods compare the unknown reading frame to related sequences, genes or motifs in proteins where direct experimental information is available. For *Blochmannia *these involve primarily various gram negative species. Thus a direct link (by sequence similarity for example) or indirect link (for example via another species) to *E.coli *proteins with experimentally verified protein function allows reliable protein function predictions in *Blochmannia*.

### Efficiency considerations

The growth in databases has resulted in a wealth of information on validated proteins and protein functions. This improves function prediction for previously not annotated (not well described) reading frames in proteobacteria. In an earlier study on the intracellular bacterium *Mycoplasma pneumoniae *[28], we estimated that five years after the original publication of the genome we could find a more accurate prediction for about a third of the reading frames. We still consider this value to be a good estimate of the benefits by database growth and algorithm development and demonstrate this here for the *Blochmannia *proteins classified as "unknown function" or "putative" in the original *Blochmannia *genome publication in 2003 [11]. For 72% of previously putative proteins a more solid function prediction could be given. This level of function reassignment would be similar for other genomes. Endosymbionts have the particular challenge that direct experimental data on expressed proteins are difficult to obtain. Conservation of a large part of their reading frames in other proteobacteria is partially helpful. On the other hand, it is well known that the conserved reading frames in *E.coli *with unclear function remain a challenge which can only be coped with by combining the most advanced techniques of sequence analysis and the additional knowledge from larger and larger data bases. Our new analysis profits from the larger database size two years later. However, this required reanalysis, all comparative sequence searches for the *Blochmannia floridanus *proteins had to be done again. Furthermore, it was important to collect and collate data from several databases as each stores other aspects of sequence function (for instance, protein families are stored in the PFAM database, clusters of orthologous groups are given in the COG database and the functional gene onthology classification is found in the GO database). We had to combine all of these different information sources to obtain the best information on the analyzed protein sequences.

### Analysis strategy

In general, we would recommend a re-consideration of the published genome sequence annotation after 3 to 5 years with a focus on the previously unknown proteins. Further reannotation steps may then follow after longer time spans. A methodological routine to re-analyze a genome sequence of interest starts with sequence comparison and analysis first, querying and collating all major sequence databases. After this there is a detailed search for molecular function. This involves protein motif searches and a study of the intrinsic features of the sequences. For the proteins which remain badly characterized in their function, genome and pathway context methods are important to establish more reliable suggestions for their function.

### *Blochmannia *protein predictions

The study illustrates that additional function assignments for proteins with previously unknown or only putative function encoded in the *Blochmannia *genome is possible combining the above techniques and ressources. In the light of the challenges to cultivate *Blochmannia *or express *Blochmannia *proteins in host cells such as *E.coli*, an analysis applying bioinformatics is an important first step to further investigate the genome and functional capabilities of this endosymbiont. Furthermore, we established links to experimentally well characterized proteins in other genomes (e.g. *E.coli*) using genome and metabolic context, the growth of databases, more powerful software and extensive sequence analysis. These analyses involved sequence similarities, however we included in addition also several other methods: Confirmation of the specific protein motifs and signatures needed for the molecular function predicted (according to experimental data from well characterized proteins of the protein family in question); functional predictions from a conserved genome context exploiting tools such as STRING [13]; the metabolic pathway context and including confirmed other proteins from the pathway to further validate the prediction; homology to structures available from structural genomics projects.

### Implications for metabolism and regulation

*Blochmannia *could be shown to be more complete in several pathways including ubiquinone synthesis, inositol and nitrogen metabolism. Furthermore, functional clusters for important cellular functions such as initiation of septum formation, for cellular regulation and replication control could be further elucidated.

From the previously 66 putative proteins 48 (72%) could now be given a clearer functional assignment. This study combines knowledge from a number of databases as well as direct sequence analysis and phylogenetic comparisons including prediction techniques for genome context [13] and pathway context [29] to establish a more complete repertoire of the exact molecular and cellular function of these *Blochmannia *proteins (Fig. 1, Fig. 2, Additional file 1, Additional file 2).

The same is true for the proteins previously classified as conserved hypothetical. The exponential growth of data, notably in bacterial genomes, and further analysis allowed specific functional prediction in 21 out of 27 cases. The present paper concisely summarizes all these predictions including pathways and functional associations of protein clusters as a basis for further research and experimental tests.

### Remaining unclear reading frames

Genes encoding proteins with unknown function conserved in several species (conserved hypothetical proteins) are currently a topic of interest, in particular regarding conserved functions contained in many bacteria, including the small endosymbiont or parasite genomes. Unfortunately, six of the conserved hypothetical proteins and, after re-examination one of the previous proteins with putative function assignment, remain unclear in their function. However, many of the *Blochmannia *proteins are conserved within proteobacteria (Table 1). This includes all just mentioned functionally unclear proteins. Among these, **Bfl310 **is so wide-spread that it occurs also in non-proteobacteria. The good conservation in several different genomes is an indicator that they present important and frequently used cellular functionalities. The putative molecular function assignments given for eleven previously conserved hypothetical proteins are confirmed by data from several databases (Additional file 2 and results). The specific molecular and cellular functions suggested for ten further proteins previously considered conserved hypothetical proteins were only made after extensive sequence analysis including comparative genomics.

## Conclusion

The data provided here by detailed sequence analysis, links to experimental data on related protein sequences as well as genome and pathway context extend the described repertoire of *Blochmannia *capabilities. This includes molecular functions for many previously conserved hypothetical or putative proteins, the nitrogen and inositol metabolism, periplasmic proteins, cell division and DNA, RNA as well as ubiquinone synthesis including some structure predictions (Fig. 3, 4, 5, results). The predicted additional functions for the studied *Blochmannia *proteins can now be further tested and analyzed. Furthermore, this study adds to the comparative characterization of the proteins considered to be the inventory of a "minimal cell" [27] looking at a compact endosymbiont genome.

## Methods

The original genome sequence and annotation [11] was reanalyzed for all conserved hypothetical or putative protein predictions. Genome context [13,26] and metabolic context [12,29] was considered and sequences and predicted pathways were extensively compared to available completely sequenced genomes to better assign and identify the encoded proteins therein. Furthermore, iterative sequence analysis compared sequences to other organisms and public databases (reviewed in [30]). The statistical expectancy value for reporting hits by chance was generally set at a conservative threshold of an expected value E of 10^-6^.

Specific sequence searches were done by applying HMMER [31]. Intrinsic sequence feature predictions were derived from the ExPASy suite of tools [32]. To independently check and test sequence analysis results, we applied not only other programs with similar function such as HMM or fasta searches, but also complementary tools and methods such as domain analysis, phylogenetic analysis, analysis of context and clusters of orthologous genes.

In addition, we applied the different tools for metabolic reconstruction and pathway alignment using extensive sequence analysis protocols as described previously [33]. Amongst other tests, this included verification of found similarities by reciprocal searches from identified sequences and determination of the exact region of sequence similarity. To delineate enzymatic capabilities, the multi-domain architecture of many proteins was taken into account: Individual parts of the protein sequence encode different domains with different functions. Sequence analysis analyzed these regions separately to identify these specific functions and the different domains in the protein. Function assignments were tested and confirmed including sequence searches from the sequence with experimentally determined function [34]. Significant links to experimentally determined function were established. Proteins classified with a high confidence (Blast e-value below 10^-6^) and informative assignment were categorized as "good" (right column; see Additional file 1). However, if there remained minor uncertainties in the function, this assignment was categorized as "fair". A protein function was classified as "putative" (15 cases; see Additional file 1), if its sequence had similarities to well characterized protein sequences or protein domains with an e-value less than or equal to 10^-3 ^and there was only a first indication on the protein function. All other cases were classified as "unknown".

Phylogenetic analysis was applied to investigate the distribution of identified proteins at different taxonomic levels (specific for *Blochmannia*, in *Enterobacteriacea*, in *Proteobacteriae*, spread among all bacteria). Further, this helped to analyze gene duplication events and to better clarify the substrate specificity of the encoded enzymes.

Further information regarding the sequence and protein family classification involved comparative genomics, gene context methods and comparisons of domains and sequences [35] including iterative searches and multiple alignments exploiting the following databanks: Clusters of orthologous groups of proteins (COGs) [36], conserved domain server [37] as well as the different protein family databases PFAM, SMART and Interpro [38].

Duplicated genes were examined further to determine which of them was the real ortholog in gene sequence comparisons [39]. Replacement by unrelated sequences (non-orthologous displacement; [40]) hampers function identification by sensitive sequence alignment procedures. In such cases, gene neighbourhood and operon context helped to determine function of reading frames. Besides this, more elaborate genome context methods were used.

**Genome context methods **and searches for functional associations exploited the STRING database [13,26]. Functional association as well as direct interaction on the protein level is predicted in the database by looking at the conservation of genome context in many different species. A first observation [14] was that reading frames which are conserved as neighbours in many genomes are a useful predictor for direct interaction of the encoded proteins. This was validated by considering proteins known to interact and the position of their reading frames. This approach allowed also to predict new interactions [14]. Subsequent studies refined genome context methods and include now also observation of gene fusion of the reading frames in one or several genomes as an even stronger predictor of interaction as well as common presence or common absence of reading frames which are functionally associated or in common pathways [26]. Furthermore, data mining (co-occurrence of genes in articles) and direct interaction data (yeast two hybrid, large scale tap-tag screens) were added as functional association indicators in the updated version of the database used for our predictions [13]. To compare and collate these different types of predictions, a prediction score is calculated, ranging from 1.0 (certain) to 0.0 (no functional association) and using Bayesian probabilities [13,26]. Four categories are distinguished [15]: Highest (0.9) and high confidence (0.7), medium confidence (0.4) and low confidence (0.15). Only the high and medium categories were used for predictions here.

**Pathway alignment **[12] compared the reading frames found to be present for a pathway of interest and a specific organism to the version present in other organisms. Sequence searches established presence of reading frames with orthologous function in better experimentally characterized prokaryotics species such as *E.coli*. These predictions were retested using biochemical data (to test for enzymes with diverged sequences escaping detection)-and calculating metabolic fluxes by elementary mode analysis (in particular to test whether missing enzyme activities are compensated by detours or alternative paths). Thresholds in the pathway alignment for sequence searches against databases were set at an expected value e below 10^-6 ^and accepted if passing the other two tests.

### Homology modelling

For several of the *Blochmannia *sequences with previously unknown function we identified homologous sequences in other species with a solved three dimensional structure. For some of these solved structures the function was not yet known as structures were solved as part of a large scale structural genomics project in that species (e.g. *E.coli, Aquifex aeolicus*). If such homologous sequences with known three dimensional structure had been identified by us, then homology models were obtained using the SWISS-MODEL server [41]. The server selects a template, creates an alignment and builds a homology models including energy minimization and WhatCheck [42] reports. Specifically, the ProModII program was used for modelling; energy minimization used Gromos96 (parameter set ifp43B1) applying steepest descent with 200 cycles and conjugate gradient with 300 cycles. The template for Bfl316 (the full *Blochmannia *sequence had 153 residues) was pdb entry 1oz9 (protein 1354 from *Aquifex aeolicus*, resolution 1.89 A). The template allowed modelling the *Blochmannia *residues 33 till 130 in the homology model shown. The template for Bfl499 (303 residues) was pdb entry 1nv8 (transferase HemK from *Methanococcus jannaschii*, resolution 1.80 A). The template allowed prediction of the residues 77 till 242 in the homology model. The template for Bfl341 was pdb entry 1ri6 (putative isomerase from *E.coli*, resolution 2.00 A). The template and the homology model obtained covered the whole Bfl341 sequence (338 residues) except the three most N-terminal residues. The root-mean-square-deviation (RMSD) for each homology model to its template was calculated. All homology model coordinates are available on request from the authors.

## Authors' contributions

All authors read and approved the manuscript and made critical comments, adding to the final version presented here. In addition they contributed

PG: Sequence comparisons, detailed bioinformatical analysis of conserved hypothetical and putative proteins

IV: Sequence comparisons, detailed bioinformatical analysis of conserved hypothetical and putative proteins

EZ: Provided experimental insights and discussion points

FJS: General analysis of genome sequence, provided phylogenetical insights and discussion points.

AM: Microbiological expertise, comparative genomics, *Blochmannia *expertise.

RG: General analysis of genome sequence, microbiological expertise, *Blochmannia *pathway and life style details

TD: Concept; lead and guided the study, bioinformatical pathway and genome context analysis.

## Supplementary Material

Additional File 3 Figure, colour drawing of the inositol pathway shown in Figure 1. Click here for file

Additional File 4 Figure, colour drawing of the nitrogen metabolism shown in Figure 2. Click here for file

Additional File 1 Table, listing the reanalyzed putative proteins. Click here for file

Additional File 2 Table, listing the analyzed conserved hypothetical proteins. Click here for file

Additional File 5 Figure, colour drawing of the homology protein model shown in Figure 3. Click here for file

Additional File 6 Figure, colour drawing of the homology protein model shown in Figure 4. Click here for file

Additional File 7 Figure, colour drawing of the homology protein model shown in Figure 5. Click here for file

## Figures and Tables

**Table 1:** Phylogenetic distribution of the previously conserved hypothetical proteins studied

Protein	*Blochmannia*	*Buchnera*/*Wigglesw.*	Proteobacteria	Bacteria general
Bfl043	+	-	+/-(no B.&W.)	-
Bfl045	+	+	+	-
Bfl048	+	-/+	+-	-
Bfl052	+	+	+	+
Bfl064 ?	+	-	+/-	-
Bfl155	+	+	+	+/-
Bfl165	+	+	+	-
Bfl220	+	+	+	+/-
Bfl248	+	+	+	-
Bfl258 ?	+	-/+	+/-	-
Bfl264	+	-	+/-(no B.&W.)	-
Bfl310 ?	+	-	+/-(no B.&W.)	+/-
Bfl316	+	+	+	-
Bfl341	+	+/-	+/-	-
Bfl363	+	-/+	+/-	-
Bfl367	+	+	+	+/-
Bfl377 ?	+	-/+	+/-	-
Bfl390	+	+	-	-
Bfl419 ?	+	-/+	+/-	-
Bfl423	+	-/+	+	+/-
Bfl442	+	+	+/-	-
Bfl451	+	+^1^	+	-
Bfl460 ?	+	+^2^/-	+/-	-
Bfl499	+	-/+	+	-
Bfl547	+	-/+	+	-
Bfl573	+	+	+/-	-

+/- indicates in proteobacteria that it did not occur in some proteobacteria, if this is missing only in *Buchnera *and *Wolbachia*, this is specifically indicated by (no B. W.).

+/- indicates in bacteria general that it did occur in some non-proteobacteria but was not wide spread. "?" indicates the protein is still unknown in its function (Bfl377 and Bfl064 can be associated with functions, see text and Additional file 2). ^1^This gene is absent in one of the three *Buchnera *genomes (BAp). ^2^This gene is absent in one of the three *Buchnera *genomes (BBp).
