# Supplementary material for: Analysis of and function predictions for previously conserved hypothetical or putative proteins in Blochmannia floridanus
Source: BMC Microbiol. 2006 Jan 9;6:1. doi: 10.1186/1471-2180-6-1 (PMC1360075; doi:10.1186/1471-2180-6-1)
Supplement: Additional File 3 — Figure, colour drawing of the inositol pathway shown in Figure 1. [file 1471-2180-6-1-S3.doc]

Malonic semialdehyde

enzymes

1D-myo-Inositol

1D-myo-Inositol-3P or D-myo-Inositol-4P or 1D-myo-Inositol-1P

Suggested to be provided by MSF transporter Bfl240, IolF-like

***Putative myo-inositol-1(or 4)-monophosphatase Bfl535***

D-2,3-Diketo 4-deoxy-epi-inositol

2-Deoxy-5-keto-D-gluconic acid

2-Deoxy-5-keto-D-gluconic acid 6-phosphat

Dihydroxyacetone phosphate

2-Inosose

***fructose-bisphosphate aldolase Bfl255***

***ADP-heptose synthase Bfl063***

***acetolactate synthase II, Bfl593, Bfl592***

Lost - probably protein of the host

Lost - probably protein of the host

***Triose phosphate isom..,Bfl601***

glyceraldehyde 3-phosphate

Putative reaction

**Additional file 3. Colour drawing of the inositol pathway shown in Figure 1.** The identified predicted enzyme activities of *Blochmannia* are mapped on the pathway of inositol synthesis. They include: a MFS family transporter (Bfl240, IolF-like, top right; exact substrate specificity not known), ADP-heptose synthase (Bfl063, IolC-like), an acetolactate synthase II, large subunit (Bfl593; IolD-like) and its small subunit, neighbouring protein Bfl 592 (both proteins should physically interact), fructose 1,6-bisphosphate aldolase (Bfl255, IolJ-like; this enzyme is also involved in glycolysis) and predicted myo-inositol-1(or 4)-monophosphatase (Bfl535; related to the Archaeal fructose-1,6-bisphosphatase and related enzymes of inositol monophosphatase family). Bfl601 (triosephosphate isomerase) finishes the pathway converting created dihydroxyacetone phosphate into glyceraldehyde 3-phosphate.
