# Supplementary material for: Analysis of and function predictions for previously conserved hypothetical or putative proteins in Blochmannia floridanus
Source: BMC Microbiol. 2006 Jan 9;6:1. doi: 10.1186/1471-2180-6-1 (PMC1360075; doi:10.1186/1471-2180-6-1)
Supplement: Additional File 4 — Figure, colour drawing of the nitrogen metabolism shown in Figure 2. [file 1471-2180-6-1-S4.doc]

Ammonia

L-Glutamine

Amides

Nitrogen

Alpha- aminoacids

Integrated in Proteins

***glutamine synthetase* Bfl618**

***beta-cystathionase* Bfl067**

***Urease cplx:***

**Bfl523, Bfl524, Bfl525**

***Associated cplx:***

**Bfl521, Bfl522 and Bfl526**

***Ferredoxin* Bfl480**

**Bfl573 *(NifU-like)***

enzymes

Putative reaction

**Additional file 4. Colour drawing of the nitrogen metabolism shown in Figure 2.** The identified previously known and newly predicted enzyme activities of *Blochmannia* are compiled for the pathway of nitrogen metabolism. They involve the urease complex (Bfl523, Bfl524 and Bfl525; catalyzing the following reaction: Urea + H_2_O = CO_2_ + 2 NH_3_) and urease activating complex (Bfl521, Bfl522 and Bfl526) and glutamine synthetase (Bfl618). Further proteins adding to the pathway are the now confirmed ferredoxin (Bfl 480), Bfl 573 (NifU-like protein; previously conserved hypothetical) as well as beta-cystathionase (Bfl067).
