# Supplementary material for: Analysis of and function predictions for previously conserved hypothetical or putative proteins in Blochmannia floridanus
Source: BMC Microbiol. 2006 Jan 9;6:1. doi: 10.1186/1471-2180-6-1 (PMC1360075; doi:10.1186/1471-2180-6-1)
Supplement: Additional File 1 — Table, listing the reanalyzed putative proteins. [file 1471-2180-6-1-S1.doc]

**Additional file 1: Table, listing of the reanalyzed putative proteins**

__________________________________________________________________________________

**Bfl protein Gene functional category molecular function assignment**^1^

**Number name**

Bfl012 *yidC* Intracellular trafficking signal transduction; good

and secretion Component:membrane

Bfl024 *pitA* Inorganic ion transport phosphate transport; good

and metabolism Component:membrane

Bfl029 *yjcE* Inorganic ion transport hydrogen antiporter activity; good

and metabolism regulation of pH; integral to

membrane

Bfl031 *yjgF* Translation Inhibits protein synthesis by good

cleavage of mRNA

Bfl036 *yjgP* inner membrane protein Predicted permeases putative

Bfl037 *yjgQ* inner membrane protein Predicted permeases putative

Bfl038 *yidZ* Transcription regulation of transcription, DNA- good

dependent

Bfl040 *Bfl040* Inorganic ion transport ABC transporter good

and metabolism

Bfl041 *Bfl041* Inorganic ion transport ABC transporter good

and metabolism

Bfl054 *yqjA* inner membrane protein inner membrane protein putative

Bfl060 *ygiH* inner membrane protein inner membrane protein putative

Bfl073 *yjeP* Cell wall/membrane Mechanosensitive ion channel; good

protection against hypo-osmotic

shock

Bfl077 *yjeE* Predicted ATPase or kinase hydrolase putative

Bfl080 *hflX* GTPases GTPases putative

Bfl090 *ytfN* Uncharacterized BCR Uncharacterized BCR unknown

Bfl134 *yabC* Cell envelope biogenesis, methyltransferase activity fair

outer membrane

Bfl180 *yfiO* DNA uptake lipoprotein DNA uptake lipoprotein good

Bfl240 *yajR* Carbohydrate transport transporter activity good

and metabolism; Amino integral to membrane

acid transport and

metabolism; Inorganic ion

transport and metabolism

Bfl250 *yqgF* Replication, recombination Predicted endonuclease involved fair

and repair in recombination

Bfl251 *yqg*E Transcription Uncharacterized ACR putative

Bfl257 *ygfA* Coenzyme metabolism 5-formyltetrahydrofolate cyclo- good

ligase family; catalytic activity

Bfl260 *ygfZ* Predicted aminomethyl- Glycine cleavage T-protein; good

transferase related to GcvT aminomethyltransferase activity

Bfl278 *yaeL* Cell envelope biogenesis, metalloendopeptidase activity good

outer membrane membrane-associated

Bfl298 *pmbA* Zn-dependent proteases Predicted modulator of DNA fair

gyrase; may be involved in

secretion;

Bfl307 *ytfF*  Carbohydrate transport and Permeases of the drug/metabolite good

metabolism; Amino acid transporter (DMT) superfamily

transport and metabolism;

Bfl318 *ubiF*  Coenzyme transport and Monooxygenase fair

metabolism, Energy

production and conversion

Bfl340 *ybgF* predicted lipoprotein predicted lipoprotein putative

Bfl343 *ybhL* inner membrane protein Integral membrane protein, good

interacts with FtsH

Bfl344 *ychF*  Translation Predicted GTPase, probable fair

translation factor

Bfl349 *hemK* Translation Methylase of polypeptide chain good

release factors

Bfl358 *sufA* HesB-like domain; HesB-like domain; unknown

may be involved in exact molecular function unknown

nitrogen fixation

Bfl359 *sufB* Posttranslational ABC-type transport system good

modification, involved in Fe-S cluster assembly,

protein turnover, chaperones permease component

Bfl374 *eaeH* putative adhesin putative adhesion putative

Bfl397 *ycfM* General function prediction Collagen-binding surface adhesin good

Only SpaP (antigen I/II family)

Bfl398 *ycfF* Nucleotide transport and Diadenosine tetraphosphate good

metabolism; Carbohydrate (Ap4A) hydrolase and other HIT

transport and metabolism; family hydrolases

Bfl399 *ycfH* Replication, recombination TatD related Dnase; DNase good

and repair enzyme;

Bfl418 *yccK* Inorganic ion transport Dissimilatory sulfite reductase good

and metabolism (desulfoviridin), gamma subunit

Bfl444 *yoaE* Inorganic ion transport involved in efflux of fair

and metabolism tellurium ions; integral to

membrane;

Bfl455 *yceL* Carbohydrate transport Arabinose efflux permease good

and metabolism

Bfl459 *gutQ* Cell wall/membrane Predicted sugar phosphate good

biogenesis isomerase involved in capsule

formation

Bfl480 *yfaE* Energy production and Ferredoxin good

conversion

Bfl521 *ureG* Posttranslational Ni_2_^+^-binding GTPase involved in good

modification, protein regulation of expression and

turnover, chaperones; maturation of urease and

Transcription hydrogenase

Bfl522 *ureF* Posttranslational UreF; nickel ion binding; nitrogen; good

modification, protein metabolism; Complex bilding with

turnover, chaperones UreG and UreD

Bfl523 *ureC* Amino acid transport and Urease alpha-subunit, N-terminal good

metabolism domain; nickel ion binding;

nitrogen metabolism;

Amidohydrolase family; hydrolase

activity

Bfl524 *ureB* Amino acid transport and Urease beta subunit; nickel ion; good

metabolism binding; nitrogen metabolism

Bfl525 *ureA* Amino acid transport and Urease gamma subunit; nickel ion good

metabolism binding; nitrogen metabolism

Bfl526 *ureD* Posttranslational UreD; nickel ion binding; nitrog good

modification, protein metabolism; Complex bilding with

turnover, chaperones UreG and UreF;

Bfl530 *engA* General function GTPase of unknown function putative

prediction only

Bfl534 *nifS* Amino acid transport Aminotransferase class-V; good

and metabolism transaminase activity; metabolism

Bfl535 *suhB* Carbohydrate transport inositol/phosphatidylinositol good

and metabolism phosphatase activity

Bfl537 *yfhC*  Nucleotide transport and Cytidine and deoxycytidylate good

metabolism; Translation deaminase zinc-binding region;

hydrolase activity

Bfl539  *pdxJ*  Coenzyme metabolism; Pyridoxal phosphate biosynthesis good

protein PdxJ; pyridoxine

biosynthesis; cytoplasm

Bfl548 *smpB* Posttranslational SmpB protein; RNA binding; good

modification, protein protein biosynthesis

turnover, chaperones

Bfl575 *yhgN* Intracellular trafficking MarC family integral membrane good

and secretion protein

Bfl576 *yigL*  General function Predicted hydrolases of the HAD putative

prediction only superfamily

Bfl578 *yigB*  General function Predicted hydrolases of the HAD putative

prediction only superfamily

Bfl606 *yibN*  Inorganic ion transport Rhodanese-related fair

and metabolism sulfurtransferases

Bfl614 *yicC*  Function unknown YicC-like family, N-terminal fair

region; play a role in stationary

phase survival; not essential during

stationary phase

Bfl615 *bfl615* Transcription MarR family; transcription factor good

activity; regulation of

transcription, repressor

Bfl620 *yigC*  Coenzyme transport 3-octaprenyl-4-hydroxybenzoate good

and metabolism carboxy-lyase; This enzyme

catalyses the third reaction in

ubiquinone biosynthesis;

Bfl623 *rumC* DNA recombination RmuC family fair

Bfl628 *yhhF* DNA replication, N6-adenine-specific methylase good

recombination and repair

_______________________________________________________________________________

^1^ *Blochmannia* protein number (the same numbers as the *Blochmannia* gene number) and gene name is followed by function assignment: For 39 proteins an informative assignment (categorized as “good”; right column) on the molecular function could be made with a high confidence (blast expected e-value below 10^-6^), for 9 proteins there remain only minor uncertainties (“fair”). For 15 proteins there is still only a putative function assignment possible and for one protein no (“unknown”) prediction is possible.
