# Supplementary material for: Analysis of and function predictions for previously conserved hypothetical or putative proteins in Blochmannia floridanus
Source: BMC Microbiol. 2006 Jan 9;6:1. doi: 10.1186/1471-2180-6-1 (PMC1360075; doi:10.1186/1471-2180-6-1)
Supplement: Additional File 2 — Table, listing the analyzed conserved hypothetical proteins. [file 1471-2180-6-1-S2.doc]

**Additional file 2: Table, listing the analyzed conserved hypothetical proteins**

**________________________________________________________________________________**

Bfl protein Gene COG e-Val Pfamdomain and existing GOs HMM remark

Number name

Bfl043 *yhbN* COG1934 1e -11 PFAM: OstA-like protein 1e-08

Bfl045 *yrbA* COG5007 3e-16 PFAM: BolA-like protein 2e-07

GO: 0030528 (transcription regulator activity)

Bfl048 *yhcB* COG3105 2e -07 PFAM: Protein of unknown function 5e -06

(DUF1043)

periplasmic membrane protein, A

assoc. with membrane transport

Bfl052 *yraL* COG0313 3e -82 PFAM: Tetrapyrrole (Corrin/Porphyrin) 2e -30

Methylases

Bfl064 *yqiC* COG2960 6e -06 PFAM: Protein of unknown function 7e -07 ?

(DUF526 – suggestion: protein biosynthesis)

Bfl155 *yadR* COG0316 4e -30 PFAM: HesB-like domain 9e -28

Bfl165 *ygbQ* COG2919 7e -12 PFAM: DivIC (Septum formation initiator) 7e -09

GO: 0007049 (cell cycle)

Bfl220 *yrdC* COG0009 1e -38 PFAM: *yrdC* domain 1e -31

Bfl248 *yggX* COG2924 5e -22 PFAM: Protein of unknown function 5e -21

(DUF495)

Bfl258 *ygfE* COG3027 1e -12 PFAM: Family of unknown function 1e -11 ?

(DUF710)

Bfl264 *ygdP* COG0494 2e -10 PFAM: NUDIX domain 7e -15

GO: 0016787 (hydrolase activity)

Bfl310 *ybeB* COG0799 3e -25 PFAM: Domain of unknown function 6e -21 ?

(DUF143– homolog of plant Iojap protein)

Bfl316 *ybeY* COG0319 3e -26 PFAM: Uncharacterized protein family 2e -19

(UPF0054)

probable membrane dependent hydrolase A

Bfl341 *ybhE* COG2706 4e -65 PFAM: no Pfam 3-carboxy muconate cyclase A  pgl (6-phosphogluconolactonase) homolog 3e-61*

Bfl363 *ynhA* COG2166 3e -30 PFAM: Fe-S metabolism associated 5e -21

domain

Bfl367 *ydhD* COG0278 5e -34 PFAM: Glutaredoxin 0,002

Bfl377 *ycaR* COG2835 5e -09 PFAM: Protein of unknown function 1e -05 ?

(DUF343- Lipopolysaccharid biosynthesis?)

Bfl390 *yqeI* COG3710 2e -12 PFAM: trans_reg_C (Transcriptional 6e -04

regulatory protein, C terminal)

GO: 0003677 (DNA binding)

0006355 (regulation of transcription,

DNA- dependent)

Bfl419 *yccV* COG3785 3e -28 PFAM: no Pfam ?

Bfl423 *ycbL* COG0491 1e -30 PFAM: Metallo-beta-lactamase 1e -15

superfamily

Bfl442 *yeaZ* COG1214 1e -37 PFAM: Peptidase_M22  2e -22

(Glycoprotease family)

GO: 0008450 (O-sialoglycoprotein

endopeptidase activity)

0006508 (proteolysis

and peptidolysis)

Bfl451 *yebA* COG0739 2e -33 PFAM: Peptidase family M23 3e -23

GO: 0004222 (metalloendopeptidase

activity)

0006508 (proteolysis and

peptidolysis)

Bfl460 *yeeX* COG2926 5e -24 PFAM: Protein of unknown function 2e -21 ?

(DUF496)

Bfl499 *yfcB* COG2890 5e -47 PFAM: no Pfam

rRNA or tRNA methylase A

Bfl547 *yfjG* COG2867 2e -18 PFAM: ARPF 5e -21

(Aromatic-Rich Protein Family)

oligoketide cyclase A

Bfl573 *yhgI* COG0694 2e -19 PFAM: HesB-like domain 7e -10

COG0316 5e -12 NifU-like domain 4e -07

_________________________________________________________________________________

*Blochmannia* protein number (the same numbers as the *Blochmannia* gene number) and gene name is followed by assignment to protein family classification systems COG ([36] e-Values given), PFAM ([31]; HMMER values are given) and GO ([44]). Remarks: “?” indicates the function is still not clear. “A” or GO indicates a protein family assignment not exactly matching the definitions of the pfam or COG classification system. *blast e-value and similarity to *E.coli* sequence *pgl*/ former *ybhE*. More functional analyses are given in the text of the results.
