# Supplementary material for: Analysis of and function predictions for previously conserved hypothetical or putative proteins in Blochmannia floridanus
Source: BMC Microbiol. 2006 Jan 9;6:1. doi: 10.1186/1471-2180-6-1 (PMC1360075; doi:10.1186/1471-2180-6-1)
Supplement: Additional File 5 — Figure, colour drawing of the homology protein model shown in Figure 3. [file 1471-2180-6-1-S5.doc]

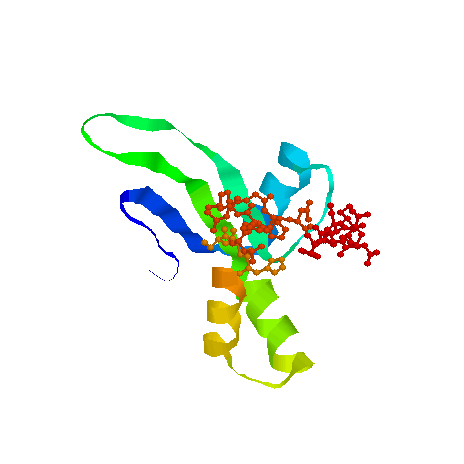


**Additional file 5. Colour drawing of the homology model of the predicted hydrolase Bfl316 shown in Figure 3.** (template:1OZ9.pdb; hypothetical protein Aq_1354 of *Aquifex aeolicus*). The model starts at residue 33, a phenylalanine. The family signature (UPF, residues from 113 to 130 in Bfl316) is indicated in ball-and-stick. Note that in the template1OZ9.pdb the structure coordinates are exactly known, however the protein function explained in results was not previously apparent.
