# Supplementary material for: Analysis of and function predictions for previously conserved hypothetical or putative proteins in Blochmannia floridanus
Source: BMC Microbiol. 2006 Jan 9;6:1. doi: 10.1186/1471-2180-6-1 (PMC1360075; doi:10.1186/1471-2180-6-1)
Supplement: Additional File 6 — Figure, colour drawing of the homology protein model shown in Figure 4. [file 1471-2180-6-1-S6.doc]

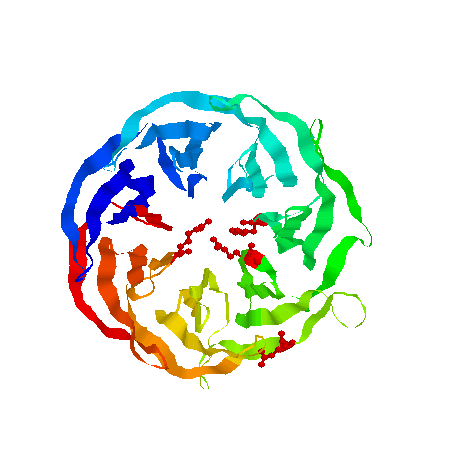


**Additional file 6. Colour drawing of the homology model of Bfl341 protein shown in Figure 4.** (template:1RI6; putative isomerase YbhE of *E. coli*)**.** The ß-propeller found in 1RI6 [24] is also evident in the homology model (starting from residue 1, a Met in Bfl341). Important predicted catalytic residues outline the substrate cleft. These are His 130, Arg 180, Glu 217 and Arg 281 of Bfl341 corresponding to His 148, Arg 196, Glu 212, and Arg 274 in the template structure (four from five conserved residues known for the family and those checked from *N.crassa* by mutagenesis to be important for catalysis in vitro [24]). Kajander et al. [24] did not know yet the accurate molecular function of the *E.coli* structure they solved. However, regarding the high sequence similarity of Bfl341 (38% identical, 57% similar residues) to recently characterized *E.coli* Pgl protein [25], the actual activity of Bfl341 we now predict to be a 6-phosphogluconolactonase.
