# Supplementary material for: Analysis of and function predictions for previously conserved hypothetical or putative proteins in Blochmannia floridanus
Source: BMC Microbiol. 2006 Jan 9;6:1. doi: 10.1186/1471-2180-6-1 (PMC1360075; doi:10.1186/1471-2180-6-1)
Supplement: Additional File 7 — Figure, colour drawing of the homology protein model shown in Figure 5. [file 1471-2180-6-1-S7.doc]

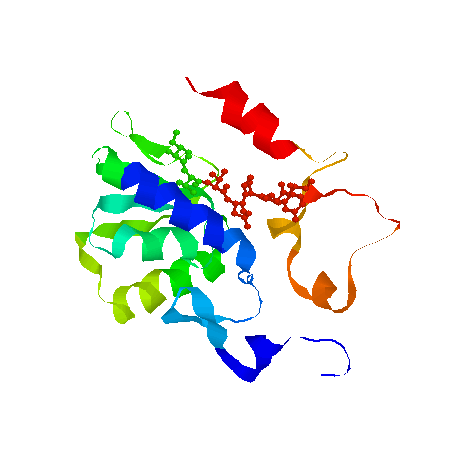


**Additional file 7. Colour drawing of the homology model of Bfl499 protein shown in Figure 5.** (template:1NV8; HemK Protein from *Metanococcus jannaschii*)**.** Sensitive sequence alignment algorithms predict this protein to be a N-6 adenine specific DNA methylase or general S-adenoslmethonine-dependent methyltransferase by similarities to experimentally well characterized proteins such as the close relative *E.coli***.** Moreover, for the homologue in *M.jannaschii* the three dimensional structure is known. This allows to establish the homology model shown here. Details of the structure: The N6-methylase prosite motif IItNPPY, residues 121 to 127, is highlighted in ball and stick. The fold consists of 3 layers, a/b/a; forming a mixed beta-sheet of 7 strands, order 3214576; strand 7 is anti-parallel to the rest. The homology of the Bfl499 protein does not include the additional helical domain present in 1NV8.
